# Computer Aided COVID-19 Diagnosis in Pandemic Era Using CNN in Chest X-ray Images

**DOI:** 10.3390/life12111709

**Published:** 2022-10-26

**Authors:** Ali Alqahtani, Mirza Mumtaz Zahoor, Rimsha Nasrullah, Aqil Fareed, Ahmad Afzaal Cheema, Abdullah Shahrose, Muhammad Irfan, Abdulmajeed Alqhatani, Abdulaziz A. Alsulami, Maryam Zaffar, Saifur Rahman

**Affiliations:** 1 Department of Networks and Communications Engineering, College of Computer Science and Information Systems, Najran University, Najran 61441, Saudi Arabia; 2 Faculty of Computer Sciences, Ibadat International University, Islamabad 44000, Pakistan; 3 Department of Computer and Information Sciences, Pakistan Institute of Engineering and Applied Sciences (PIEAS), Islamabad 44000, Pakistan; 4 Electrical Engineering Department, College of Engineering, Najran University, Najran 61441, Saudi Arabia; 5 Department of Information Systems, College of Computer Science and Information Systems, Najran University, Najran 61441, Saudi Arabia; 6 Department of Information Systems, Faculty of Computing and Information Technology, King Abdulaziz University, Jeddah 21589, Saudi Arabia

**Keywords:** COVID-19 pandemic, contact tracing, CNN, chest X-ray images, hybrid learning, machine learning, computer-aided diagnosis

## Abstract

Early detection of abnormalities in chest X-rays is essential for COVID-19 diagnosis and analysis. It can be effective for controlling pandemic spread by contact tracing, as well as for effective treatment of COVID-19 infection. In the proposed work, we presented a deep hybrid learning-based framework for the detection of COVID-19 using chest X-ray images. We developed a novel computationally light and optimized deep Convolutional Neural Networks (CNNs) based framework for chest X-ray analysis. We proposed a new COV-Net to learn COVID-specific patterns from chest X-rays and employed several machine learning classifiers to enhance the discrimination power of the presented framework. Systematic exploitation of max-pooling operations facilitates the proposed COV-Net in learning the boundaries of infected patterns in chest X-rays and helps for multi-class classification of two diverse infection types along with normal images. The proposed framework has been evaluated on a publicly available benchmark dataset containing X-ray images of coronavirus-infected, pneumonia-infected, and normal patients. The empirical performance of the proposed method with developed COV-Net and support vector machine is compared with the state-of-the-art deep models which show that the proposed deep hybrid learning-based method achieves 96.69% recall, 96.72% precision, 96.73% accuracy, and 96.71% F-score. For multi-class classification and binary classification of COVID-19 and pneumonia, the proposed model achieved 99.21% recall, 99.22% precision, 99.21% F-score, and 99.23% accuracy.

## 1. Introduction

The first case of viral disease COVID-19 [1] was registered in December 2019 in China’s city Wuhan, which was subsequently proclaimed in March 2020 as a pandemic by WHO (World Health Organization). Coronavirus is also recognized as SARS-CoV- [2]. It is from the same group as MERS-CoV and SARS-CoV, which were discovered in 2003 & 2015, respectively [3]. As stated by the European Centre for Disease Prevention and Control [4], on 29 July 2021 about 34,435,890 cases were reported as positive for COVID-19, out of which 743,712 deaths were reported. It affects almost every aspect of life including health, education, the economy, etc. A wide part of employees lost their livelihoods due to this outbreak. There are no proper medicines or vaccines discovered yet. However, it can be controlled to some extent through early detection. One of the most common and widely used techniques is RT-PCR [5], which is a real-time detection method. RT-PCR samples are collected through a swab that is inserted into the nose and mouth of the patient to collect the samples. These samples are then sent to labs for testing, but it is a complex, time-consuming manual practice. Automatic detection is an alternative method recommended for the early detection of coronavirus.

Early studies show that chest radiograph images of patients who are infected with coronavirus illustrate some irregularities. The easy availability and accessibility of these radiograph images in areas with limited resources make them better options than PCR [6,7]. However, to examine these radiograph images to detect the infected areas of the lungs, experienced and skilled radiologists are required, but the computer-aided diagnosis system CADx also solves this problem for radiologists. It detects the presence of the virus that causes COVID-19 quickly and with high precision. CADx, using chest X-rays, should have been designed to fight against this virus [8]. Machine learning (ML) and deep learning (DL) play a vital role in the medical field for the detection and treatment of many of the deadliest diseases like brain tumors, chest cancer, etc. During the last couple of decades, deep learning showed vast progress in terms of efficient and accurate predictions. Due to this great ability of generalization, it is able to solve many complex problems of computer vision like image classification, organ detection, disease identification, etc. [9,10].

Deep learning is an advanced field and is a further subclass of machine learning. Its CNN algorithm gives far better results than any other traditional algorithm. One of the best features of CNN is that it automatically extracts features from the images without using handy craft filters. Sometimes, parameter learning through a limited dataset can cause overfitting problems. This problem can be tackled by using pre-trained architectures of CNN like GoogleNet, DenseNet, and VGG-16, which are trained on the ImageNet datasets. By using the transfer learning technique, we can apply the pre-trained architectures to our limited dataset. For this purpose, we have to remove the last few layers of the pre-trained model and then test it on our specific dataset. Proper hyper-parameters and efficient fine-tuning make it a more effective approach [11,12].

In this study, we proposed a COV-Net architecture-based computer-aided diagnosis system for COVID-19 analysis. In the presented work, we used the benchmark dataset which contains images of chest X-rays of viral pneumonia-infected patients, COVID-19 patients, and normal images to train our proposed COV-Net model. In D-HL, boundary homogeneity-related deep features are extracted from the fully connected layer FC-1 of the proposed COV-Net architecture. For structural risk minimization and to enhance the generalization ability of the proposed framework, we used SVM as a classifier for the final prediction. The proposed COV-Net contains four convolutional blocks, and optimized arrangements of layers facilitate better and more efficient learning with fewer parameters as compared to state-of-the-art deep CNNs. The following are the contributions of this study:

1. A new, well fine-tuned CNN architecture named COV-Net with fewer parameters is proposed to diagnose COVID-19 efficiently.
2. Using edges exploitation operation in an optimized structure with the convolutional operator facilitates learning edges-related features of infection patterns in chest X-ray images. It leads to improved detection of COVID-19 in a timely manner.
3. A D-HL-based framework for COVID-19 and pneumonia identification in chest X-ray images was proposed by using new deep CNN and SVM.
4. We exploit the structural and empirical risk error minimization using the proposed COV-Net and ML classifier in hybrid learning (HL) for COVID-19 analysis. In the proposed deep hybrid learning scheme, the learning capability of the proposed CNN is explored and ML classifiers are used to enhance the discrimination proficiency of the proposed framework for chest X-ray analysis.

## 2. Related Work

Many experiments have been conducted in the last two years to recognize the viral disease COVID-19 by using deep learning methods and traditional machine learning techniques. L. Lin et al. [13] applied a framework that proposed CNN with ResNet50 as its backbone, which extracts 2D as well as 3D features from CT images and then combines them through max-pooling followed by the softmax function, which gives an AUC equal to 0.96. X. Xu et al. [14] devoted classic ResNet architecture to differentiate coronavirus from I-AVP. The model along with the location-attention mechanism provided 86.7% accuracy. 

G. Biraja et al. [15] conducted a study to determine uncertainty using drop-weights based on BCNNs. He used a pre-trained model Resnet50-V2 with fully connected layers then applied drop-weights followed by a softmax layer. It gained an accuracy of 89.92%. W. Shuai et al. [16] exploited both static and dynamic data for the detection of patients with the potential to move from a malignant to a critical stage. Static data included a personal and clinical record of the person while a series of CT images served as dynamic data. They combined static data with dynamic and fed them to MLP, which then served as input for the long short-term memory (LSTM). J. Cheng et al. [17] designed a model which classifies four categories including COVID-19, influenza A/B, CAP, and non-pneumonia patients by using the U-Net-34 2D segmentation network. Resent-152 is the backbone of the 2D classification deep learning network and achieved 94.98% accuracy with an area under an AUC of 97.71. J. Shuo et al. [18] created and installed an AI system within four weeks for the detection of COVID-19 to reduce the burden on radiologists and clinicians.

They used UNet++ for lung segmentation of CT images along with ResNet50 and got a specificity of 0.922 and sensitivity of 0.974. N. Ali et al. [19] performed three different types of two-class classifications with four different classes (viral pneumonia, COVID-19, bacterial pneumonia, and normal). Due to the limited availability of the dataset, the transfer learning technique was used, which uses five pre-trained DL architectures: ResNet101, ResNet52, ResNet50, Inception- ResNet-V2, and Inception. Resnet50 showed the best results for all three binary classifications (classification 1: 96.1%, classification 2: 99.3%, classification 3: 99.7%). They also applied approaches with and without pre-training of COVID-CAP, and achieved 95.7% and 98.3% accuracy, respectively. L. Wang et al. [20] created a dataset called COVIDx containing 13,975 chest X-beam images and used the model COVID-Net for recognition of COVID-19, obtaining an accuracy of 92.4%. M. Abed Mohammed et al. [21] associated deep learning models (like DarkNet, GoogleNet, ResNet50, MobileNets V2, and Xception) and traditional ML models (like KNN, decision tree, ANN, SVM with linear kernel, and RBF), and results demonstrated that DL frameworks outperformed ML frameworks and achieved 98.8% accuracy with ResNet50 architecture, while ML model SVM achieved its best accuracy of 95% and 94% with RBF kernel. D.Hemdan et al. [22] used the COVIDX-Net framework which includes seven different CNN models (Visual Geometry Group Network (VGG19), Inception-ResNet-V2, DenseNet121, InceptionV3, ResNetV2, Xception, and MobileNetV2) to categorize COVID-19 negative or positive cases. Architectures VGG19 and DenseNet121 gave almost similar results for the detection of normal and COVID-19 and gave F1-scores of 0.89 and 0.91, respectively. 

A. Khandakar et al. [23] developed a vigorous method for the automatic recognition of coronavirus and pneumonia from chest X-ray scans and used pre-trained DL models to maximize the accuracy of detection. H.S. Maghdid et al. [24] purposed a simple CNN model to detect COVID-19 for early diagnosis. V. Chauhan et al. [25] applied transfer learning methodology and used pre-trained models to extract features. The results of pre-trained models were combined with a prediction vector and majority voting was used for the final prediction. T. Rahman et al. [26] have proposed three different schemes of classifications: normal/pneumonia classification, bacterial/viral pneumonia classification, and normal/bacterial/viral pneumonia classification. M. Loey et al. [27] exploited the transfer learning technique with GAN to detect coronavirus using chest X-rays. GAN helped in decreasing the overfitting issue produced by the small dataset and increased the dataset to 30 times more than the original dataset. A. Degerli et al. [28] proposed a novel strategy that not only detects coronavirus but also quantifies the severity by creating infection maps. They tried two configurations: first, they froze the encoder layers, then they permitted them to fluctuate. A. O. Ibrahim et al. [29] proposed an automatic DL structure for coronavirus-infected areas. They trained and tested the proposed model to check the effectiveness and generalization by using slices of 2D CT.

Generally reported work in literature lakes advocated the following points:

1. Most of the work presented in the past has been assessed using only accuracy, but recall, precision, and F-score are better performance measures to evaluate the generalization of the model for the complex dataset.
2. In most of the previous works, only COVID-19 detection is performed. However, simply detecting COVID-19 is insufficient to diagnose other severe abnormalities, e.g., pneumonia.
3. In COVID-19 analysis, the detection rate of infected X-ray images from normal individuals is still challenging because of fewer inter-class variations.

To overcome these limitations, we proposed a multi-class chest X-ray classification method using standardized performance evaluation matrices like recall, precision, F-score, and accuracy for improved diagnosis. 

## 3. Methods and Materials

**Figure 1:**
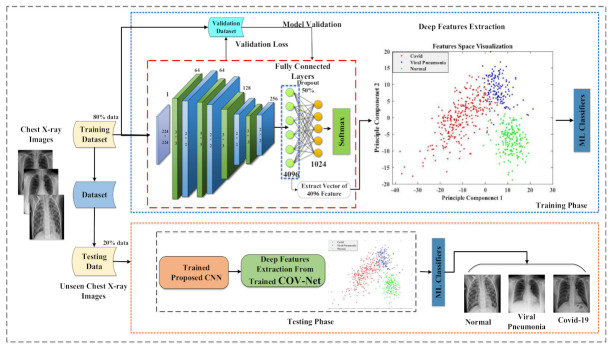
Block-based figure of proposed COVID-19 analysis model.

In the proposed work, COVID-19 detection was performed by using the proposed COV-Net CNN and the ML classifier and included some phases. First, X-ray images went through the preprocessing pipeline, which included data augmentation. At that point, a preprocessed dataset was split into training and testing datasets. We trained our proposed COV-Net-based model by using a training dataset. Training accuracy and loss were computed after every epoch. Testing data were used to evaluate the performance of the proposed method by following the appraisal metrics of accuracy, precision, recall, and F-score. A detailed overview of the proposed methodology is demonstrated in Figure 1.

### 3.1. Dataset

**Figure 2:**
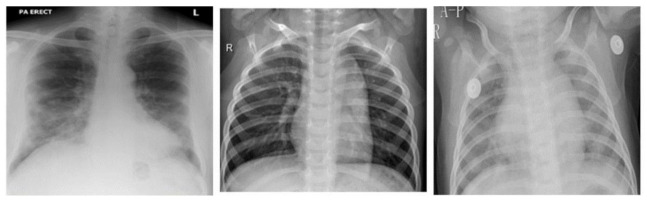
Sample images from dataset of three classes (normal, COVID-19, pneumonia).

In this work, we used the chest X-rays dataset. From the dataset, 300 normal chest X-ray pictures, 300 images of viral pneumonia, and only 300 images of coronavirus-infected patients were selected. All images were collected from the publicly available Kaggle repository [30]. The exhibition of the framework greatly depended upon the accuracy of the dataset. For this reason, we first sampled the data before using them. In data sampling, we only used those images that were useful and eliminated falsified images. The dataset contained chest X-rays of three classes (COVID-19/pneumonia/normal). All images are in JPEJ format as shown in Figure 2; the first one shows a normal chest X-ray, the second one shows a COVID-19 X-ray, & the third one shows a pneumonia X-ray.

### 3.2. Data Augmentation

Data augmentation is a method to increase the data samples during the training of the model. After employing data balancing and sampling, we contained 300 images of each class for better and generalized model training. We applied the data augmentation method to enhance and increase the dataset instances for better training of the model and to avoid overfitting [31]. Different data augmentation methods were applied in random rotation and random horizontal translation, as described in Table 1, which yielded an augmented dataset batch during training of the proposed model.

### 3.3. Proposed CNN Architecture

**Figure 3:**
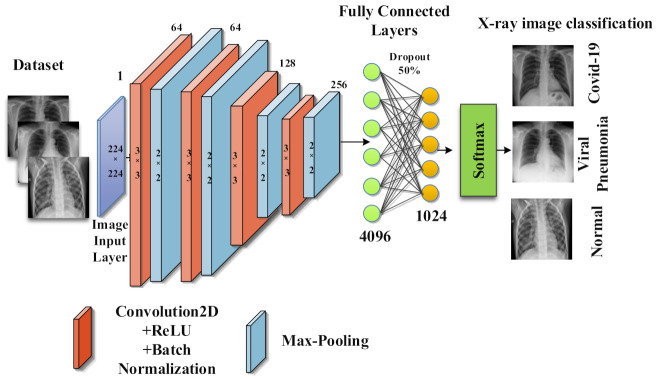
Detailed overview of proposed COV-Net.

The proposed CNN architecture COV-Net used in this study included four convolutional blocks. Each block was constituted of a convolutional layer, batch normalization, and activation function, namely ReLU, followed by max-pooling as shown in Figure 3. In convolutional layers, filters convolved over the input image. The convolutional function performed the dot product of filter and valued and extracted features from the input images. CNN used a backpropagation algorithm for dynamic feature extraction. One of the advantages of CNN over ANN is that it automatically extracts domain-specific features from the images. By further using an edge operator (max-pooling), it learned profoundly discriminative features to train the model. In the pooling, layer down-sampling was also performed, which enhanced the performance of the model by making a small variation in the input image and by decreasing the non-linear dimensions of the resulting feature maps.

**Figure 4:**
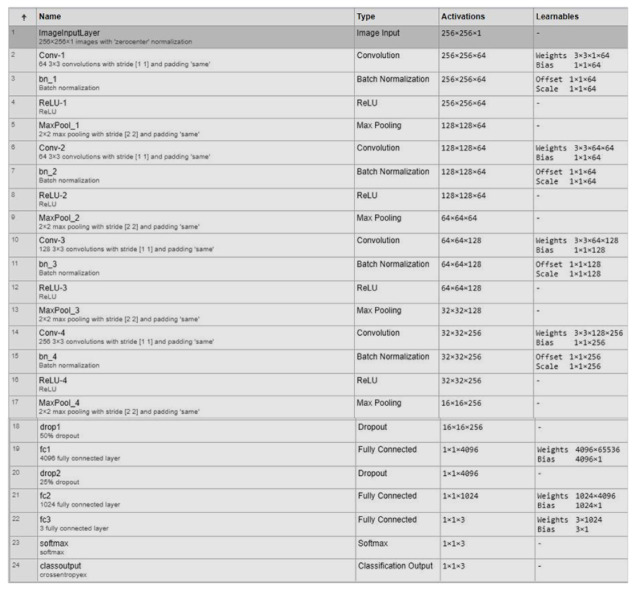
Architectural detail of proposed COV-Net.

To highlight the features for classification, resulting feature maps were extracted from a fully connected layer. A dropout layer was added at the end to avoid overfitting. Detailedd layer wise description of propsed imodel is illusterated in Figure 4. The cross-entropy function was used as a cost function along with the softmax function. To categorize COVID-19, healthy people, and viral pneumonia, we used traditional ML classifiers, namely, random forest, Naïve Bayes, support vector machine (SVM), k-Nearest Neighbor (k-NN), and ensemble model.

In this study, we used MATLAB to run the code. In the training phase of our proposed COV-Net model, we used the “rmsprop” function as an optimizer. It is a gradient-based method. It normalized the gradient by balancing the momentum, diminishing the progression for a large gradient to obtain from exploding, and expanding the progression for a small gradient to obtain from vanishing [32]. After an experimental analysis, an optimal learning rate of “0.0001” was selected. To reduce computational complexity, the batch size was set to 16 per epoch, which is a small size. To improve generalization, L1 regularization was used. As a cost function, the cross-entropy function was used along with the softmax function.

### 3.4. Implementation Details

The “RMSPROP” function was used as an optimizer. In the beginning, “0.0001” learning rate was selected randomly and 50 epochs were used, meaning each photo of training data was examined 50 times. As only 50 epochs were selected due to the limited dataset, we chose a large number of epoch models to move towards overfitting, which means instead of training, the model started removing the available small dataset. For this reason, we chose to lose many epochs to avoid overfitting. 

### 3.5. Initial Training

We split the data into two parts: training and testing; 80% of them were used for training while 20% were set aside for testing according to Pareto’s Principle [33]. We saved 10% of the 80% of the training dataset for validation, and the remaining 70% was utilized to train the model. Initial training helped us to check what our model can yield as a baseline model. Before starting training of the model, many different preprocessing techniques were used to boost the performance of the model. As we proposed, a CNN model was used so the training starts from scratch. In our proposed COV-Net model, we used the softmax function along with the cross-entropy cost function for classification.

### 3.6. Feature Extraction Using Proposed CNN Architecture

**Figure 5:**
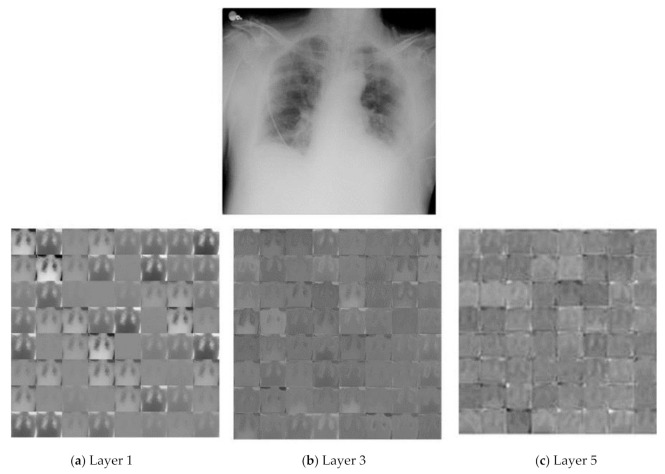
Features maps representation from three different layers of proposed COVID-Net architecture, (**a**) layer1, (**b**) layer3, and (**c**) layer5.

In the proposed work, we proposed new CNN architecture to obtain deep features from chest X-rays. The proposed CNN architecture extracts the most discriminative and deep features. The first fully connected layer (FC-1) extracted 4096 features from the images, which we used as a feature vector. Figure 5 shows the resulting feature maps from various layers of the CNN model of sample images of chest X-rays.

At the primary level, almost complete data that are present in the input image are saved by activations.

(a) As we go to the higher layer, activation started to keep fewer data.
(b) At a deep level, the information became more detailed.

The uprising of the data into a more detailed and higher level was associated with each layer of the proposed CNN COV-Net (the deeper the network, the more composite the data and information). The proposed architecture COV-Net extracted features from input images. We extracted 4096 highlights from the FC-1 layer, and these highlight vectors were fed into different conventional machine learning classifiers as input to discover if the inspected patient was positive for COVID-19, viral pneumonia-infected, or just a normal patient. The dynamic features we used in our proposed model were driven by the FC-1 layer as shown in Table 2.

### 3.7. Classification Using Conventional ML Classifiers

The proposed CNN COV-Net architecture was used to extract features from the augmented dataset. We extracted features from the FC-1 layer, and details are shown in Table 2. After extracting the features, these features were passed as input to conventional ML classifiers to train them. Different ML classifiers like Naïve Bayes, decision trees, KNN, and SVM determine the robustness of the classification. The performance of these models was measured by classifying COVID-19, pneumonia-infected, and healthy patients. The accuracy of classification attained by using conventional ML classifiers performed better than the softmax function. This is because it extracted the most highlighted features from chest X-rays of different patients by using the most abstract feature extraction techniques.

#### 3.7.1. SVM

SVM is a linear model. It can tackle linear and non-linear issues. Its basic idea is that it makes a line to separate two classes. New data components are assigned to one class based on predictive analysis. As a rule, a parallel classifier expects that the data being referred to contain two potential objective variables. It utilizes a procedure called kernel trick to change the data and then find boundaries between them. It groups data and trains models inside really limited levels of extremity, making a three-dimensional order model that simply follows the X/Y prescient axis [34].

(1) $$L\left(\gamma ,\alpha ,\beta \right)=\frac{1}{2}∥\gamma {∥}^{2}-\sum \sum_{i=1}^{m}\beta i\left[\mathrm{yi}\left(\gamma \cdot x+\alpha \right)1\right]$$

#### 3.7.2. k_NN

This is used for regression as well as classification. Its calculation utilizes highlight closeness to anticipate the upsides of any new information focuses, implying that the new point is allocated a worth dependent on how intently it resembles training dataset points [32].

(2) $$\sqrt{\overset{n}{\underset{i=1}{\sum }}{\left(qi-pi\right)}^{2\;}}$$

#### 3.7.3. Naïve Bayes

It is a group of algorithms that are based on the “Bayes Theorem”. They work on the principle that every pair of classifying features is independent [35].

(3) $$P\left(U|V\right)=\frac{P\left(V|U\right)P\left(U\right)}{P\left(V\right)}$$

#### 3.7.4. Random Forest

It is fundamentally a supervised method. It is an ensemble model which contains multiple decision trees. It collects results from all decision trees and then, based on the highest voting, makes a decision [36].

(4) $$RFfi_{i}=\frac{\sum j\in all\;trees\;normfi_{ij}\;}{T}$$

### 3.8. Performance Metrics

Classification performance of the model is calculated through different performance metrics, for example, accuracy [37], recall [38], precision [38], and F-score [39], etc. When classifying medical images, we use different terms like false negative, false positive, etc.

#### 3.8.1. Precision

It is the proportion of correct positive predictions to the total positive prediction. It indicates the rate of correct positive predictions. It is calculated as:

(5) $$Precision=\frac{TP\;}{\left(TP+FP\right)\;\;\;\;\;}\times 100$$

#### 3.8.2. Recall

In this, we calculate true positive predictions from total positive predictions that might have been made. It shows a number of missing positive predictions. It is calculated as:

(6) $$Recall=\frac{TP\;}{\left(TP+FN\right)\;\;\;\;\;}\times 100$$

#### 3.8.3. Accuracy

It is the most regular performance measure. It gives correct predictions to the total predictions.

(7) $$Accuracy=\frac{\left(TN+TP\right)}{\left(FP+FN+TP+TN\right)}$$

#### 3.8.4. F-Score

It shows steadiness between recall and precision.

(8) $$F-score=\frac{2\times \left(Precision+Recall\right)\;\;}{Precision+Recall}$$

## 4. Results

In our research, we presented a CNN model which extracted features from the augmented dataset. We had a small dataset, so we applied the data augmentation method to enhance the dataset. The augmented dataset also played an important part in accuracy improvement because of its high generalization ability. The proposed model was trained with 50 epochs under a batch size of 8. Deep and discriminative features were extracted from the proposed CNN architectures. The extracted features were passed as input to some conventional ML classifiers, e.g., Naïve Bayes, KNN, random forest, and support vector machine. In the event of binary classification of COVID-19 and pneumonia, KNN and SVM achieved 100% accuracy, recall, precision, and F1-score, shown in Table 3 and Table 4.

We also evaluated our proposed D-HL method with the baseline proposed COV-Net to emphasize the performance improvement of our proposed method. Table 5 proved that our proposed technique enhanced the discrimination strength of our proposed model in accuracy (1.73%) and F-score (1.68%).

## 5. Discussion

In the presented D-HL architecture, the softmax layer was replaced with a machine learning classifier. The CNN learning algorithm utilized empirical risk minimization as a method to reduce false positives and false negatives during training. When the back-propagation algorithm reaches the first hyperplane that separates, the training phase ends, and progress generally stops as a result. Another limitation of CNN is that it frequently assigns one output neuron a high value (around +1) while assigning low values to the other neurons (close to 1).

This makes it very difficult to reject implementation errors. Softmax classifiers provide us with likelihoods for each class label. On the other hand, conventional ML techniques help us develop a robust rejection strategy. The generalization ability of CNN is weaker compared to that of SVM DL approaches, in contrast to conventional ML methods, are the least understandable from an AI aspect and are assumed to as a black box.

**Figure 6:**
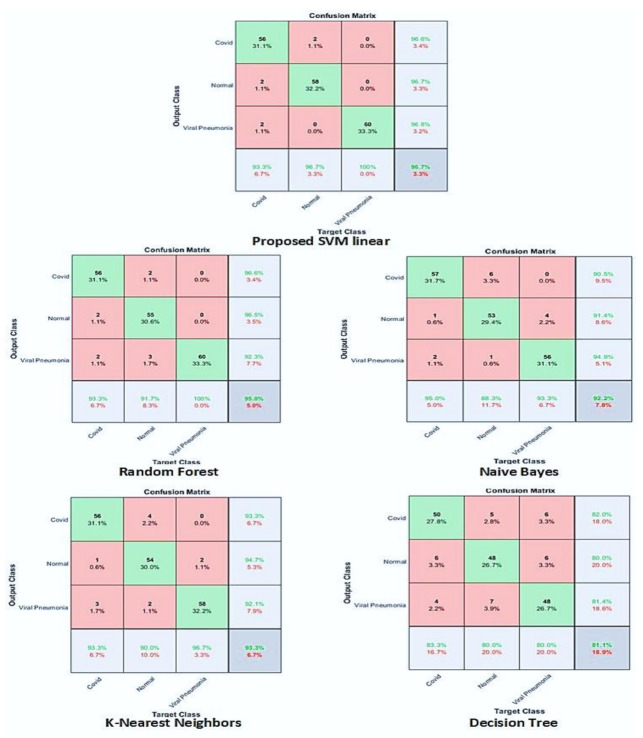
Confusion matrix-based performance analysis of competitive ML classifiers.

We performed classification with three classes as well as with two classes. In three classes, pneumonia, normal, and COVID-19 were included and in binary classifications, we used COVID-19 and pneumonia. SVM gave the highest accuracy with three classes. We achieved outstanding accuracy in binary classification with SVM and KNN. In the case of three classes, SVM gave an accuracy of 96.7%, recall of 96.6%, precision equal to 96.7%, and F1-score equal to 96.7%. Table 3 shows the detailed overview of the proposed CNN architecture with all four conventional ML classifiers. Confusion matrixes based on the performance analysis of classifiers are demonstrated in Figure 5. We compared results obtained by the proposed method with other existing works based on different performance metrics. Apostolopoulos et al. [40] used five different CNN pre-trained architectures to classify between three classes(COVID-19, normal, and pneumonia) and gave a sensitivity of 98.66%, accuracy of 94.72%, and specificity of 96.46%. H.S. Maghdid et al. [24] used the transfer learning technique with the AlexNet model and got 94.1% accuracy, 72% sensitivity, and 100% specificity. S.S Khan et al. [41] applied a convolutional auto-encoder to achieve 0.7652 area under a curve. A. Narin et al. [19] also used five CNN models (ResNet50, ResNet101, ResNet152, inception-ResNetV2, and InceptionV3) to perform binary classification of four classes and achieve an accuracy of 96.1%, recall of 91.8%, specificity of 96.6%, F1-score of 83.5%, and precision of 76.5% with COVID-19 & normal binary classification. R. Kumar et al. [42] performed an experiment with DenseNet & GoogleNet and attained an F-score equal to 0.91, AUC: 0.97. Similarly, Makris A. et al. [43,44] used five different pre-trained CNN models and achieved 95% accuracy. Arora, R. et al [45] proposed stochastic deep learning model using ensemble of slandered convolutional models and evaluate developed model on standard dataset contain three classes: COVID-19, normal and pneumonia and attain an accuracy and AUC of 0.91 and 0.97, respectively. A detailed comparison is illustrated in Figure 6 and Table 6.

**Figure 7:**
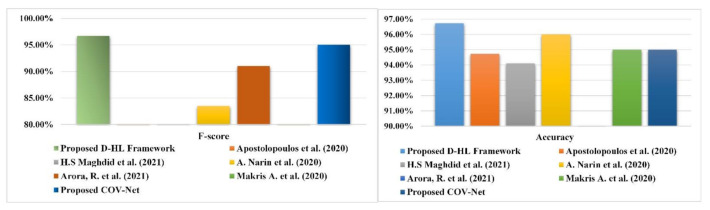
Comparative analysis of proposed COV-Net and D-HL with existing literature using accuracy and F-score [19,24,40,43,45].

Experimental results show that our proposed models outperform all these experiments and achieved 96.69% recall, 96.72% precision, 96.73% accuracy, and 96.71% F-score, as shown in Table 5 and Figure 7.

Certain limitations still apply to our research investigation. The training period for feature extraction was lengthy due to the tiny batch sizes employed to extract the runtime features, which would have typically required a large amount of GPU RAM. Second, the proposed framework must go through a thorough clinical trial before radiologists’ professional judgment may be utilized to resolve the patient data.

## 6. Conclusions

Well-timed identification of COVID-19 infection is vital to preserve the patient’s life and control the further spread of this life-threatening disease. In this study, a new CNN-based scheme for the detection of COVID-19 is proposed. COVID-19 analysis is performed using chest X-ray images containing three categories (pneumonia, COVID-19, and normal). Experimental results proved that the hybrid learning-based framework has shown improved performance compared to other methods. When the proposed framework’s performance is compared with the state-of-the-art deep models’, it shows that the proposed deep hybrid learning-based method achieved 96.69% recall, 96.72% precision, 96.73% accuracy, and 96.71% F-score for multi-class classification, and for COVID-19 and pneumonia we achieved 99.21% recall, 99.22% precision, 99.21% F-score, and 99.23% accuracy. The proposed COV-Net is less complex than pre-trained and custom-designed networks, and it is feasible to run it on ordinary current PCs. This is conceivable because the algorithm requires fewer resources for both training and execution. Performance analysis is carried out to attain the generalized model and it is likely to assist radiologists in making decisions in their clinical practice.

## Figures and Tables

**Table 1:** Augmentation parameter details.

Parameters	Values
Random Rotation	[−5, 5]
Random Horizontal Translation	[−0.5, 1]
Random Vertical Translation	[−0.5, 1]

**Table 2:** Extracted features detail of proposed architecture.

Features Layer	Feature Dimension
FC-1	1 × 1 × 4096

**Table 3:** Performance comparison using ML classifiers for two classes (Pne = pneumonia, Cov = COVID-19). The Bold shows results of proposed method.

Classifiers	Parameters	Type	TP	FP	FN	Recall (%)	Precision (%)	F1-Score (%)	Accuracy (%)
KNN	K = 2	Cov	60	0	0	100	100	100	100
		Pne	60	0	0				
	K = 3	Cov	60	0	0	100	100	100	100
		Pne	60	0	0				
	K = 4	Cov	60	0	0	100	100	100	100
		Pne	60	0	0				
	K = 5	Cov	59	1	0	99.2	99.2	99.2	99.2
		Pne	60	0	1				
SVM	**Linear**	**Cov**	**60**	**0**	**1**	**99.21**	**99.22**	**99.21**	**99.23**
		**Pne**	**59**	**1**	**0**				
	RBF	Cov	60	0	1	99.2	96.2	97.7	99.2
		Pne	59	1	0				
	Gaussian	Cov	60	0	1	99.2	99.2	99.2	99.2
		Pne	50	1	0				
	PolyOrder-2	Cov	60	0	0	100	100	100	100
		Pne	60	0	0				
	PolyOrder-3	Cov	60	0	1	99.2	99.2	99.2	99.2
		Pne	59	1	0				
	PolyOrder-4	Cov	60	0	0	100	100	100	100
		Pne	60	0	0				
	PolyOrder-5	Cov	60	0	0	100	100	100	100
		Pne	60	0	0				
Decision tree		Cov	55	5	1	95	95.2	95.1	95.0
		Pne	59	1	5				
Naïve Bayes		Cov	59	1	1	98.3	98.3	98.3	98.3
		Pne	59	1	1				
RF	max no. of splits 5	Cov	59	1	0	99.15	99.2	99.2	99.2
		Pne	60	0	1				

**Table 4:** Performance comparison of proposed framework using ML classifiers for three classes (Nor = normal, Cov = COVID-19, Pne = pneumonia). The Bold shows results of proposed method.

Classifiers	Parameters	Type	TP	FP	FN	Recall (%)	Precision (%)	F1-Score (%)	Accuracy (%)
K-Nearest Neighbors	K = 2	Cov	56	4	5	92.2	92.2	92.2	92.2
		Pne	56	4	4				
		Nor	54	6	5				
	K = 3	Cov	56	3	3	93.3	93.3	93.3	93.3
		Pne	58	2	5				
		Nor	54	6	3				
	K = 4	Cov	57	3	5	93.3	93.3	93.3	93.3
		Pne	58	2	4				
		Nor	53	7	3				
	K = 5	Cov	53	7	3	92.2	92.4	92.3	92.2
		Pne	58	2	8				
		Nor	55	5	3				
Decision Tree		Cov	50	10	11	81.1	81.1	81.1	81.1
		Pne	48	12	11				
		Nor	48	12	12				
Naïve Bayes		Cov	57	3	6	92.2	92.3	92.2	92.2
		Pne	56	4	3				
		Nor	53	7	5				
Random Forest	max no. of splits 5	Cov	56	4	2	95	95.1	95.1	95.0
		Pne	60	0	5				
		Nor	55	5	2				
SVM	**Linear**	**Cov**	**56**	**4**	**2**	**96.6**	**96.7**	**96.7**	**96.7**
		**Pne**	**60**	**0**	**2**				
		**Nor**	**58**	**2**	**2**				
	Gaussian	Cov	57	3	5	94.5	94.5	94.5	94.4
		Pne	58	2	3				
		Nor	55	5	2				
	RBF	Cov	56	4	5	93.9	93.9	93.9	93.9
		Pne	58	2	3				
		Nor	55	5	3				
	Poly- Order3	Cov	57	3	3	95.6	95.6	95.6	95.6
		Pne	60	0	3				
		Nor	55	5	2				
	Poly- Order4	Cov	57	3	3	95	95.1	95.03	95.0
		Pne	60	0	4				
		Nor	54	6	2				
	Poly- Order5	Cov	56	4	3	94.43	94.5	94.5	94.4
		Pne	60	0	5				
		Nor	54	6	2				

**Table 5:** Proposed hybrid learning method comparison with proposed COV-Net.

Model	Recall	Precision	Accuracy	F-Score
Proposed COV-Net	95.0%	95.07%	95.0%	95.03%
Proposed D-HL-based Framework	96.69%	96.72%	96.73%	96.71%

**Table 6:** Proposed hybrid learning method comparison with existing techniques on publicly available dataset. The Bold shows results of proposed method.

Author	Methodology	Recall	Precision	Accuracy	F-score
Apostolopoulos et al. (2020) [40]	VGG19, MobileNet, Inception, Xception, Inception ResNet v2.	98.6%	-	94.72%	-
H.S Maghdid et al. (2021) [24]	Transfer learning with AlexNet model	72%	-	94.1%	-
A. Narin et al. (2020) [19]	Pre-trained CNN architectures: ResNet50, ResNet101, ResNet152, inception-ResNetV2 and InceptionV3	91.8%	76.5%	96%	83.5%
Arora, R. et al. (2021) [45]	CNN architecture DenseNet & GoogleNet	91%	-	-	91%
Makris A. et al. (2020) [43]	5 pre-trained CNNs	-	-	95%	-
**Proposed DH-L** **Framework**	**Proposed COV-Net with conventional ML classifier**	**96.69%**	**96.72%**	**96.73%**	**96.71%**

## Data Availability

Data are available in publicly accessible repositories which are described in Section 3.1.
